# Smoking and job satisfaction among electrical technicians

**DOI:** 10.1192/j.eurpsy.2025.995

**Published:** 2025-08-26

**Authors:** A. Abbes, I. Sellami, A. Feki, M. A. Ghrab, M. Hajjaji, S. Baklouti, M. L. Masmoudi, K. Jmal Hammami

**Affiliations:** 1 Occupationnal medecine; 2 Rheumatology, CHU Hedi Chaker, Sfax, Tunisia

## Abstract

**Introduction:**

To ensure the health of employees, maintaining a smoke-free workplace is essential. Understanding the relationship between smoking and work is crucial for implementing effective smoking cessation interventions.

**Objectives:**

Our study aims to determine the prevalence of smoking and its relationship with job satisfaction among electrical technicians.

**Methods:**

We conducted a descriptive, analytical and cross-sectional survey among electrical technicians. Data collection was carried out using a self-completed questionnaire. We collected socio-professional data. Nicotine dependence was assessed using the Fagerström test, while job satisfaction was evaluated using the single-item measure of job satisfaction.

**Results:**

Our study population was exclusively male, including 70 electrical technicians. The mean age of participants was 38.1 ±10.2 years. The mean of the tenure of job was 14.5±11.1 years. Active smoking was reported by 45.1% of participants. Using the Fagerström test, nicotine dependence was low and moderate to high respectively in 13.4% and 28.9% of smokers. Median job satisfaction was 4, with extreme values ranging from 1 to 5. We found that nicotine dependence was negatively correlated with job satisfaction (p = 0.02, r = -0.3).

**Conclusions:**

Smoking is a prevalent issue among electrical technicians. Our findings highlight the urgent need for smoking cessation interventions among these workers. The association of smoking with low job satisfaction underscores the importance of preventive measures to reduce work-related stress in order to promote greater job satisfaction and smoking cessation.

**Disclosure of Interest:**

None Declared

